# Long noncoding RNA ZFAS1 promotes gastric cancer cells proliferation by epigenetically repressing KLF2 and NKD2 expression

**DOI:** 10.18632/oncotarget.9611

**Published:** 2016-05-26

**Authors:** Fengqi Nie, Xiang Yu, Mingde Huang, Yunfei Wang, Min Xie, Hongwei Ma, Zhaoxia Wang, Wei De, Ming Sun

**Affiliations:** ^1^ Department of Oncology, Second Affiliated Hospital, Nanjing Medical University, Nanjing, People's Republic of China; ^2^ Department of General Surgery, The Affiliated Yantai Yuhuangding Hospital of Qingdao University Medical College, Yantai, People's Republic of China; ^3^ Department of Medical Oncology, Huai'an First People's Hospital, Nanjing Medical University, Huai'an, People's Republic of China; ^4^ Department of Bioinformatics and Computational Biology, UT MD Anderson Cancer Center, Houston, TX, USA; ^5^ Department of Biochemistry and Molecular Biology, Nanjing Medical University, Nanjing, People's Republic of China; ^6^ Department of Pathology, First Affiliated Hospital of Nanjing Medical University, Nanjing, People's Republic of China

**Keywords:** long noncoding RNA, ZFAS1, gastric cancer, proliferation, KLF2 and NKD2

## Abstract

Recently, long noncoding RNAs have been emerged as critical regulators of human disease and prognostic markers in several cancers, including gastric cancer. In this study, we globally assessed the transcriptomic differences of lncRNAs in gastric cancer using publicly available microarray data from Gene Expression Omnibus (GEO) and identified an oncogenetic lncRNA ZFAS1, which may promote gastric tumorigenesis. ZFAS1 has been found to be upregulated and function as oncogene in colorectal cancer and hepatocellular carcinoma, but its expression pattern, biological function and underlying mechanism in gastric cancer is still undetermined. Here, we reported that ZFAS1 expression is also overexpressed in gastric cancer, and its increased level is associated with poor prognosis and shorter survival. Knockdown of ZFAS1 impaired gastric cancer cells proliferation and induced apoptosis in vitro, and inhibited tumorigenicity of gastric cancer cells in vivo. Mechanistically, RNA immunoprecipitation and RNA pull-down experiment showed that ZFAS1 could simultaneously interact with EZH2 and LSD1/CoREST to repress underlying targets KLF2 and NKD2 transcription. In addition, rescue experiments determined that ZFAS1 oncogenic function is partly dependent on repressing KLF2 and NKD2. Taken together, our findings illuminate how ZFAS1 over-expression confers an oncogenic function in gastric cancer.

## INTRODUCTION

Gastric cancer is one of the leading causes of cancer related death worldwide, and is the most common gastrointestinal malignancy in East Asia [1, 2]. In spite of the improvement in surgical techniques and targeted drug chemotherapy, the five-years overall survival rate remains unsatisfactory due to lots of patients were diagnosed at an advanced stage accompanied by lymphatic metastasis that limit the successful therapeutic strategies [3]. Although there are a great advancement on the gastric carcinogenesis, the molecular mechanisms underlying gastric cancer development and progression are still poorly understood [4]. Therefore, better understanding of the tumorgenesis is essential for the development of diagnostic markers and aid novel effective therapies for gastric cancer patients.

In the past decade, compliance of human genome sequencing and GENCODE project has revealed that only less than 3% of human genome are protein coding genes, while the major of the rest is noncoding genes yielding lots of noncoding transcripts including microRNAs and long non-coding RNAs (lncRNAs) [5, 6]. lncRNAs are a class of ncRNA that greater than 200 nt in length, with no potential protein translation capacity. Several studies have documented that lncRNAs participate in multiple biological process including imprinting, epigenetic regulation, alternative splicing, RNA decay, cell differentiation, cell cycle control, cancer cells metastasis and drug resistance [7–9]. In addition, lncRNA expression is frequently dysregulated in human disease, and few specific lncRNAs are associated with cancer cells metastasis and patients poor prognosis [10–12]. Therefore, lncRNAs have been highlighted as key players in cancer research, and lots of studies have revealed that lncRNAs may function as tumor suppressors, oncogenes or both depending on the circumstance.

Recently, several lncRNAs have been reported to involved in gastric tumorigenesis and progression, such as HOTAIR, ANRIL, BC032469, HOXA-AS2 et al [13–16]. LncRNA BC032469 expresses highly in gastric cancer, and promotes cells proliferation by function as competing endogenous RNA for miR-1207-5p and antagonizing its repression on hTERT [17]. LINC00152 overexpression facilitates gastric cancer cell proliferation through accelerating the cell cycle by binding to EZH2 and repressing p15 and p21 transcription [18]. Our previous studies showed that increased HOXA-AS2 promotes gastric cancer cells proliferation by epigenetically silencing P21/PLK3/DDIT3 expression [15], and HOTAIR function as oncogene through acting as a competing endogenous RNA for miR-331-3P or repressing miR34a by binding to PRC2 to promote the epithelial-to-mesenchymal transition in gastric cancer [13, 19].

ZFAS1 is a newly identified lncRNA that is downregulated in human breast cancer, which may serve as a tumor suppressor [20]. However, recent studies showed ZFAS1 amplification in HCC and CRC. ZFAS1 interacts with CDK1 and involves in p53-dependent cell cycle control and apoptosis in CRC cells and promotes HCC cells metastasis by binding miR-150 and abrogating its tumor-suppressive function [21, 22]. However, the expression pattern, biological roles and underlying molecular mechanisms of ZFAS1 in gastric tumorigenesis remain unclearly defined. In this study, we found that ZFAS1 expression is upregulated in gastric cancer tissue and cell lines by analyzing publicly available microarray data from GEO and validating in an cohort of 54 pairs tissue samples. Moreover, in vitro and in vivo loss- and gain-of function assays were performed to investigate the roles of ZFAS1 on regulating gastric cancer cell phenotypes, and mechanism investigations document by which mechanism ZFAS1 regulating its underlying targets.

## RESULTS

### lncRNA ZFAS1 is overexpressed in gastric cancer tissues and cell lines

**Figure 1:**
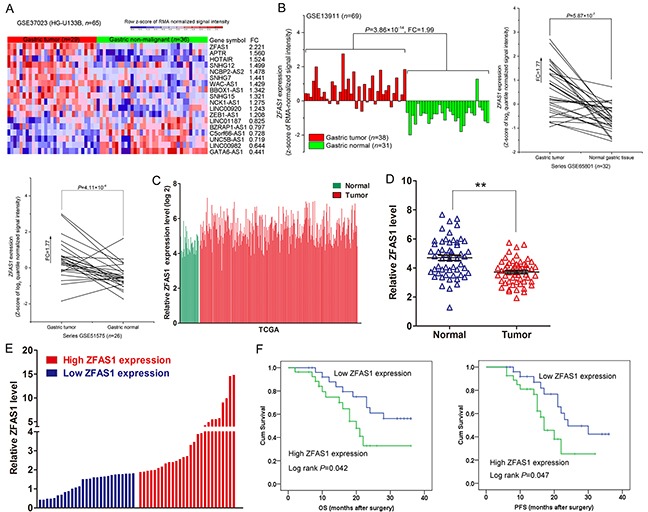
ZFAS1 expression is upregulated in NSCLC tissues and associated with poor prognosis **A, B**. Relative expression of ZFAS1 in gastric cancer tissues compared with normal tissue was analyzed by using GEO datasets including GSE37023, GSE13911, GSE65801 and GSE51575. **C**. Relative expression of ZFAS1 in gastric cancer tissues compared with normal tissue was analyzed by using TCGA data. **D**. ZFAS1 expression level in gastric cancer tissues (n = 54) compared with corresponding non-tumor tissues (n = 54) was examined by qPCR and normalized to GAPDH expression. The data were presented as the delta CT value. **E**. The patients were classified into two groups according to ZFAS1 expression. **F**. Kaplan–Meier analysis of three years overall survival and disease-free survival according to ZFAS1 expression levels. *P < 0.05, **P < 0.01.

To identify lncRNAs involved in gastric cancer development and progression, four microarray datasets (GSE37023, GSE13911,GSE65801 and GSE51575) were obtained from GEO to analyze lncRNAs alterations between gastric cancer and pair nontumor tissues. Analysis of these data showed that lncRNA ZFAS1 was the most up-regulated lncRNA in GSE37023 dataset (Figure 1A), and ZFAS1 expression was also consistently up-regulated in GSE13911, GSE65801 and GSE51575 datasets (Figure 1B). In addition, analysis of TCGA stomach and normal tissues RNA sequencing data also showed that ZFAS1 expression is up-regulated in tumor tissues compared with normal tissues (Figure 1C). To validate the analysis finding, we detected ZFAS1 expression in an cohort of 54 pair gastric cancer and normal tissues, and 5 gastric cancer cell lines. The results confirmed that ZFAS1 expression is increased in gastric cancer tissues and cells (Figure 1D and Supplementary Figure S1A). Therefore, we speculate that ZFAS1 may function as an important oncogene in gastric cancer.

### ZFAS1 amplification is correlated with poor prognosis in gastric cancer

To explore the relationship between ZFAS1 expression and gastric cancer clinicopathologic, the patients were divide into two groups: the high ZFAS1 group (n=27, fold-change ≥median), and the low ZFAS1 group (n=27, fold-change ≤median) (Figure 1E). Statistical analysis revealed that increased ZFAS1 expression were correlated with tumor size (p = 0.014), and advanced pathological stage (P=0.001). However, ZFAS1 expression was not associated with other factors including gender (p = 0.207) and age (p = 0.500) in gastric cancer (Table 1). Next, we conducted a Kaplan–Meier survival analysis to explore the correlation between ZFAS1 expression and gastric cancer patients' prognosis. The results showed that patients with higher ZFAS1 levels had a shorter overall survival (OS) and Progression-free survival (PFS) time than those with low ZFAS1 levels (Figure 1F). These data suggest that ZFAS1 is up-regulated and correlated with poor prognosis in gastric cancer.

**Table 1:** Correlation between ZFAS1 expression and clinicopathological characteristics of gastric cancer patients (n=54)

Characteristics		ZFAS1		P value
		Low	High	
**Age**	<50	12	13	0.500
	>50	15	14	
**Gender**	Male	11	15	0.207
	Fmale	16	12	
**location**	Distal	12	7	0.314
	Middle	10	15	
	Proximal	5	5	
**Tumor size**	<5cm	17	8	0.014*
	>5cm	10	19	
**Histologic**	Well	1	2	0.05*
	Moderately	12	4	
	Poorly	9	18	
	Undifferentiated	5	3	
**Lymphatic metastasis**	NO	11	15	0.207
	YES	16	12	
**TNM stages**	I	6	0	0.001*
	II	13	5	
	III	8	20	
	IV	0	2	

^*^ Overall P<0.05

### ZFAS1 promotes gastric cancer cells proliferation in vitro

**Figure 2:**
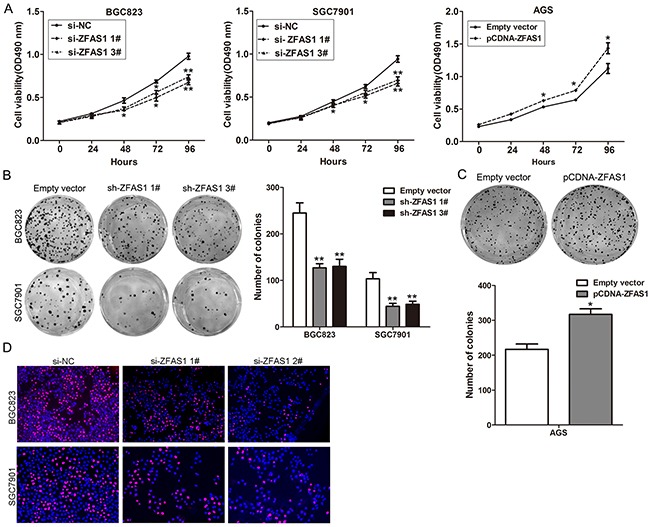
Effects of ZFAS1 on gastric cancer cell proliferation in vitro **A**. MTT assays were performed to determine the cell viability for si-ZFAS1-transfected BGC823, SGC7901cells and pCDNA-ZFAS1 transfected AGS cells. **B, C**. Colony-forming assays were used to determine the colon ability of si-ZFAS1-transfected BGC823, SGC7901 and pCDNA-ZFAS1 transfected AGS cells. **D**. EdU staining assays were performed to determine the growth of si-ZFAS1-transfected BGC823 and SGC791 cells. All experiments were performed in biological triplicates. *P<0.05, **P<0.01.

To investigated the biological function of ZFAS1 in gastric cancer cells, ZFAS1 expression was knockdown in BGC823 and SGC7901 cells by transfection with siRNAs or shRNA vector, and over-expressed by transfected with pCDNA-ZFAS1 vector (Supplementary Figure S1B–S1D). To assess the role of ZFAS1 in gastric cancer cells phenotype, we performed loss-of-function and gain-of-function assays. MTT assays showed that growth of BGC823 and SGC7901 cells transfected with si-ZFAS1 was impaired compared with control cells, while ZFAS1 ovexpression promoted AGS cells proliferation (Figure 2A). Moreover, colony formation assays showed that ZFAS1 knockdown impaired GC cells colon ability, while ZFAS1 ovexpression increased AGS cells colon formation ability (Figure 2B–2C). EdU staining assays showed the same results (Figure 2D).

### Knockdown of ZFAS1 induced gastric cancer cells apoptosis

**Figure 3:**
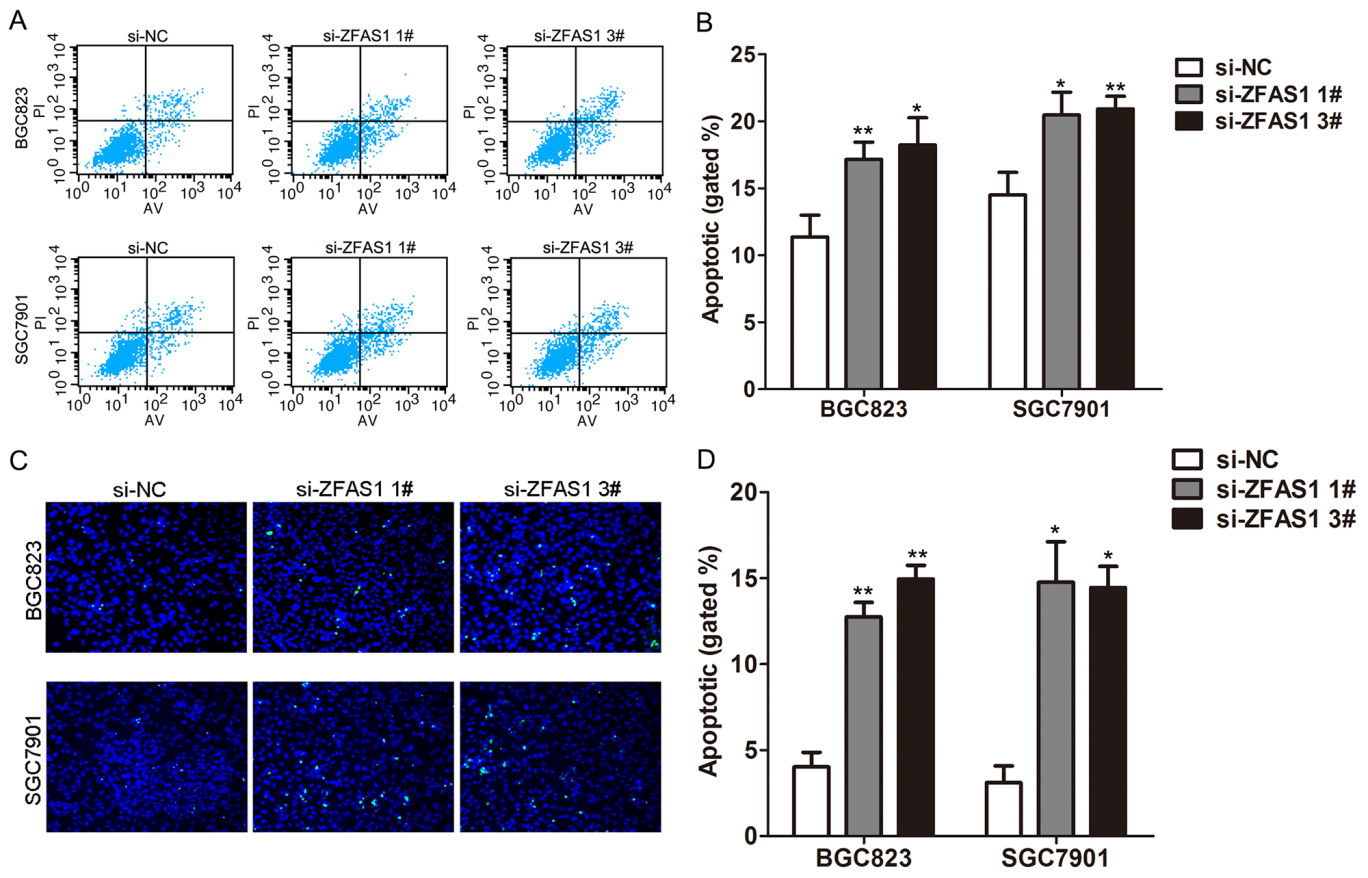
Knockdown of ZFAS1 induced gastric cancer cell apoptosis **A, B**. Flow cytometry cell apoptosis assays were used to analyze the cell apoptotic in si-ZFAS1 transfected BGC823 and SGC7901cells. **C, D**. Tunel staining assays were conducted to analyze the cell apoptotic of BGC823 and SGC7901 cells after transfected with si-LINC01133. Data are presented as mean ± SE. All experiments were performed in biological triplicates. *P<0.05, **P<0.01.

To further determine whether the effect of ZFAS1 on gastric cancer cells proliferation reflected cell apoptosis, we performed flow cytometry and Tunel staining assays. The results showed that BGC823 and SGC7901 cells transfected with ZFAS1 siRNA had higher apoptotic rate in comparison with control cells (Figure 3A–3D). These data indicate that ZFAS1 could promote the proliferation phenotype and inhibit apoptosis of gastric cancer cells.

### Knockdown of ZFAS1 inhibits gastric cancer cell tumorigenesis in vivo

**Figure 4:**
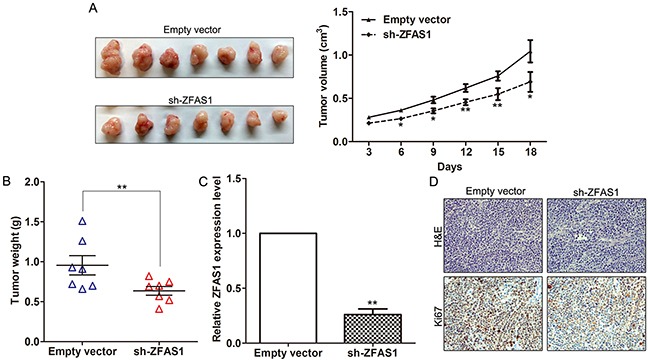
Effect of ZFAS1 on gastric cancer cells tumorigenesis in vivo **A**. The stable ZFAS1 knockdown BGC823 cells were used for the in vivo assays. The tumors from two groups nude mice were shown and tumor growth curves were measured and shown after the injection of BGC823 cells. The tumor volume was calculated every 3 days. **B**. Tumor weights from two groups are represented. **C**. ZFAS1 expression level in tumor tissues formed from BGC823/sh-ZFAS1 and BGC823/empty vector was detected by qPCR. **D**. Tumors developed from sh-ZFAS1 transfected BGC823 cells showed lower Ki67 protein levels than tumors developed from control cells. Upper: H & E staining; Lower: immunostaining. *P<0.05, **P<0.01.

To further investigate whether knockdown of ZFAS1 expression could affect tumor growth in vivo, BGC823 cells stably transfected with sh-ZFAS1 or empty vectors were inoculated into male nude mice. Eighteen days after injection, the tumor size in the sh-ZFAS1 group was significantly smaller compared with the control group (Figure 4A). Moreover, AGS cells stably transfected with ZFAS1 vector or empty vector was inoculated into male nude mice. Eighteen days after injection, the tumor size in the ZFAS1 vector group was larger compared with the control group (Supplementary Figure S2A). The tumor weight of sh-ZFAS1 group was also significantly lower than that in the control group (Figure 4B). Next, qPCR assays determined that ZFAS1 expression levels were down-regulated in tumor tissues collected from sh-ZFAS1 group compared with control (Figure 4C). Moreover, immunohistochemistry (IHC) analysis confirmed that the tumors formed from BGC823/sh-ZFAS1 cells displayed lower Ki-67 staining than those formed from the control cells (Figure 4D). Our results indicated that knockdown of ZFAS1 expression could suppress gastric cancer cells tumor growth in vivo.

### ZFAS1 directly binds with EZH2, LSD1/CoREST in gastric cancer cells

**Figure 5:**
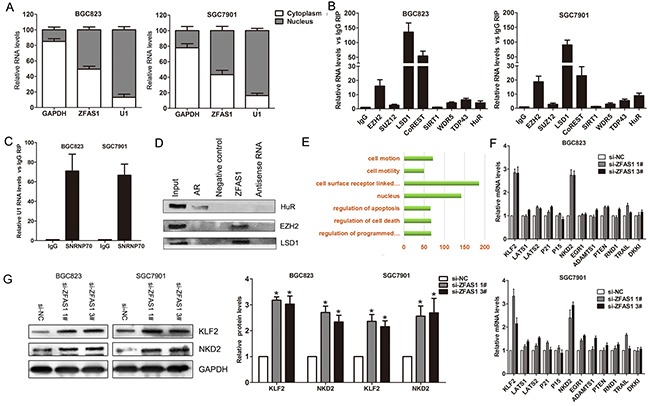
ZFAS1 interacted with EZH2 and LSD1/CoRET, and regulate KLF2 and NKD2 expression **A**. Relative ZFAS1 levels in BGC823 and SGC7901 cell cytoplasm or Nucleus were detected by qPCR. GAPDH was used as cytoplasm control and U1 was used as nuclear control. **B**. ZFAS1RNA levels in immunoprecipitates with EZH2, SUZ12, LSD1 et al. antibodies were determined by qPCR. The expression levels of ZFAS1 RNA were presented as fold enrichment relative to IgG. **C**. SNRNP70 RNA levels in immunoprecipitates with U1 antibodies were used as positive control. **D**. EZH2 and LSD1 protein levels in immunoprecipitates with ZFAS1 RNA were determined by western blot. HuR protein immunoprecipitates with AR RNA was used as positive control. **E**. Pathways analysis of ZFAS1 associated genes in gastric cancer tissues. **F**. The levels of KLF2, LATS1/2, P21, P15, and NKD2 et al. mRNA were determined by qPCR when knockdown of ZFAS1 in BGC823 and SGC7901 cells. **G**. The KLF2 and NKD2 protein levels were determined by western blot in ZFAS1 knockdown BGC823 and SGC7901 cells. All experiments were performed in biological triplicates. *P<0.05, **P<0.01.

Generally, lncRNAs regulate their target genes expression through interacting with RNA binding proteins such as PRC2 or acting as endogenous competing RNAs for miRNAs et al. To investigate the molecular mechanism of ZFAS1 involved in gastric cancer cells, we firstly analysis the distribution of ZFAS1 in gastric cancer cells. The results showed that ZFAS1 is distributed in both cytoplasm and nucleus, but the ratio of ZFAS1 in nucleus is more higher (Figure 5A). Furthermore, we performed RIP assays and the results showed that ZFAS1 could directly binds with EZH2, LSD1 and CoREST in BGC823 and SGC7901 cells (Figure 5B), while U1 binding with SNRNP70 was used as positive control (Figure 5C). In addition, RNA-pulldown assays confirmed that ZFAS1 indeed binds with EZH2 and LSD1 in BGC823 cells (Figure 5D). These data suggest that ZFAS1 could epigenetically repress underlying targets expression at transcriptional level.

### NKD2 and KLF2 are key downstream mediator of ZFAS1 in gastric cancer cells

To further explore the underlying target genes of ZFAS1 in gastric cancer cells, we constructed gene coexpression networks using TCGA data to find the ZFAS1 associated genes. Coexpression network analysis showed that ZFAS1 related genes involved in several pathways including regulating of cell apoptosis and death, and cell motility (Figure 5E). Among of these genes, we chose some important tumor suppressors to determine which one could be ZFAS1 target. The qPCR results showed that ZFAS1 knockdown increased the expression of KLF2 and NKD2, but not others (Figure 5F). Similarly, western blot analysis showed the same results (Figure 5G).

**Figure 6:**
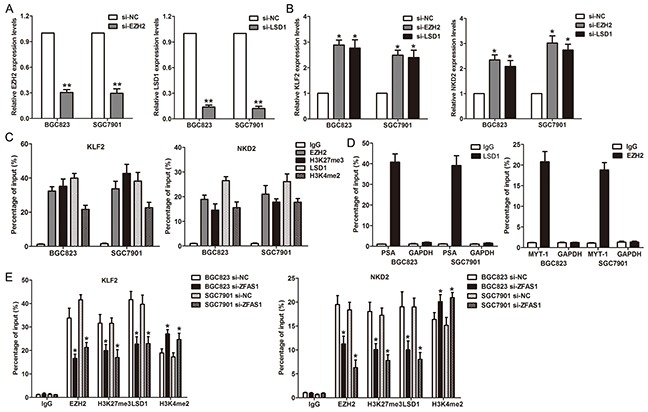
ZFAS1 recruits EZH2/ LSD1 to KLF2 and NKD2 promoter and represses their transcription **A**. The EZH2 and LSD1 expression levels were detected by qPCR when knockdown of EZH2 or LSD1 in BGC823 and SGC7901 cells. **B**. The KLF2 and NKD2 expression levels were detected by qPCR when knockdown of EZH2 or LSD1 in BGC823 and SGC7901 cells. **C**. ChIP–qPCR analysis of LSD1and EZH2 occupancy, H3K4me2 and H3K27me3 binding in the KLF2 or NKD2 promoter in BGC823 and SGC7901 cells, and IgG as a negative control. **D**. ChIP–qPCR analysis of LSD1 occupancy in the PSA, and EZH2 occupancy in the MYT-1 promoter in BGC823 and SGC7901 cells, which was used as positive control. **E**. ChIP–qPCR analysis of LSD1and EZH2 occupancy, H3K4me2 and H3K27me3 binding in the KLF2 or NKD2 promoter after knockdown of ZFAS1. All experiments were performed in biological triplicates.*P<0.05, **P<0.01.

To determine whether ZFAS1 repressed NKD2 and KLF2 expression via interacting with EZH2 or LSD1 in gastric cancer cells, we evaluated their expression after knockdown of EZH2 and LSD1 in gastric cancer cells. Interestingly, either knockdown of EZH2 or LSD1 up-regulated KLF2 and NKD2 expression (Figure 6A–6B). To confirm whether EZH2 or LSD1 could directly bind the promoter region of KLF2 and NKD2, we designed four pairs of primers across 2000 bp of the promoter region. ChIP assays showed that EZH2 and LSD1 could bind to the KLF2 and NKD2 promoter region (Figure 6C–6D). Moreover, knockdown of ZFAS1 reduced their binding to KLF2 or NKD2 promoter regions (Figure 6E). Finally, correlation analysis revealed that ZFAS1 expression is inversely correlated with KLF2 and NKD2 levels in 20 paired gastric cancer tissues (Supplementary Figure S2C)

### Silencing of KLF2 and NKD2 is partly involved in the oncogenic function of ZFAS1

**Figure 7:**
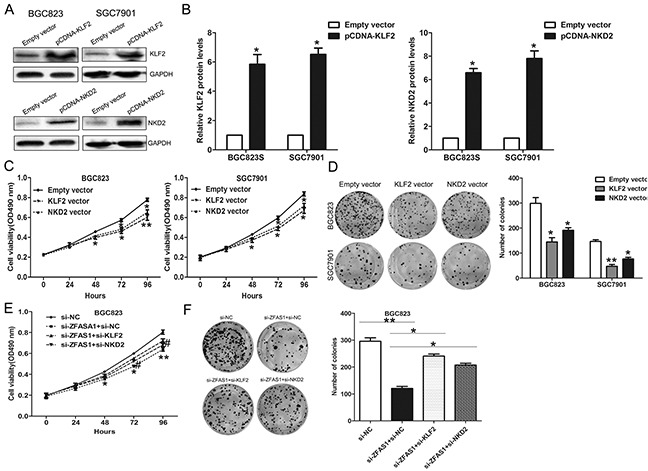
ZFAS1 function as oncogene by repressing NKD2 and KLF2 expression in gastric cancer cells BGC823 and SGC7901 cells were transfected with pCDNA-KLF2, pCDNA-NKD2, or co-transfected with si-ZFAS1, si-KLF2 or si-NKD2. **A, B**. The protein level of NKD2 and KLF2 was detected by western blot. **C, D**. MTT assays and colony-forming assays were performed to determine the cell viability. Values represent the mean ± S.E from three independent experiments. **E, F**. MTT and colony-forming assays were used to determine the cell viability. *P < 0.05 and **P < 0.01

To further determine whether KLF2 and NKD2 is involved in the ZFAS1 induced promotion of gastric cancer cells proliferation, we detected their expression in 20 pairs gastric cancer and normal tissues and found that their expression are both decreased in gastric cancer tissues (Supplementary Figure S2B). Next, we performed gain-of-function assays, and the western blot assays confirmed that KLF2 and NKD2 expression was significantly up-regulated in BGC823 cells transfected with pCDNA-KLF2 and pCDNA-NKD2 compared with control cells (Figure 7A–7B). MTT and colon formation assays demonstrated that cell proliferation was inhibited upon overexpression of KLF2 and NKD2 (Figure 7C–7D). Moreover, we conducted rescue assays to determine whether KLF2 and NKD2 involved in ZFAS1 contributions to gastric cancer cell proliferation. BGC823 cells were co-transfected with si-ZFAS1 and si-KLF2 or si-NKD2, and si-KLF2 or si-NKD2 transfection could partly rescue si-ZFAS1 decreased cells growth (Figure 7E–7F). These findings indicate that ZFAS1 exerting oncogenic effects in gastric cancer cells may partly through repressing KLF2 and NKD2 expression.

## DISCUSSION

Hundreds of lncRNAs in human cancers have been discovered by RNA-sequencing of several types of cancers sample and stored in databases such as TCGA [23]. Hence, lncRNAs have been emerged as critical regulators of gene expression and key players in cancer cells, and a growing interest toward lncRNAs in cancer is sparked [24]. For example, lncRNA-NUTF2P3-001 induced by hypoxia contributes to tumorigenesis of pancreatic cancer through derepressing the miR-3923/KRAS pathway [25]. Antti et al. established PCAT5 as a novel oncogenic lncRNA in ERG positive prostate cancers by deep transcriptome sequencing [26]. In addition, many lncRNAs have been found to involve in gastric tumorigenesis and progression, such as HOTAIR [13], H19 [27], MEG3 [28], HOXA-AS2 [15] and KRT7-AS [29]. In this study, we identified ZFAS1, a frequently amplified lncRNA, as an oncogenic lncRNA in gastric cancer by analyzing microarray data from GEO. Validation in an cohort of 54 pairs gastric cancer and normal tissues revealed that ZFAS1 is upregulated in gastric cancer tissues and correlated with poor prognosis and shorter survival. Knockdown of ZFAS1 impaired gastric cancer cells growth and induced cell apoptosis in vitro and inhibited tumorigenesis of gastric cancer cells in vivo.

Generally, lncRNA involved in regulation of cancer cells phenotypes through regulating target gene expression by different mechanisms, including chromatin modification, genomic imprinting, RNA decay and sponging miRNAs. In previous study, ZFAS1 was reported to act as an ceRNA in HCC cells by sponging miR-150 and derepressing its regulation of ZEB1, MMP14, and MMP16 expression [21]. Whether ZFAS1 could regulate underlying targets expression through other mechanisms in cancer cells is undermined. Interestingly, we found that ZFAS1 could directly bind with EZH2, LSD1 and CoREST (histone demethyltransferase of REST complex) in gastric cancer cells, which suggesting that ZFAS1 might also could regulate underlying targets at transcriptional levels. Further investigations determined that tumor suppressors NKD2 and KLF2 are novel ZFAS1 targets in gastric cancer cells. ZFAS1 simultaneously recruits EZH2 and LSD1 to NDK2 and KLF2 promoter region and represses their transcription via mediating trimethylation of histone H3 at lysine 27 (H3K27me3) and demethylation of H3K4me2. These findings indicated that lncRNA ZFAS1 play key roles in EZH2 and LSD1 mediated repression of tumor suppressors in gastric cancer cells.

KLF2 is an member of the Kruppel-like factor (KLF) family that with Cys2/His2 zinc-finger domains [30]. There is evidence showed that its expression is diminished in multiple cancers and possesses tumor-suppressor features for its inhibitory effects on cell proliferation [31, 32], and our previous study showed that SUZ12 could repress its expression in gastric cancer cells [33]. In this study, we also found that KLF2 can function as tumor suppressor and its' expression could be suppressed by ZFAS1 through recruiting EZH2 and LSD1 to its promoter region in gastric cancer cells. NKD2, one of the naked cuticle (NKD) family, is frequently methylated and suppresses proliferation by inhibiting Wnt signaling in human breast cancer [34]. In addition, NKD2 suppresses tumor growth and metastasis in osteosarcoma by function as a negative regulator of Wnt signaling, which is critical for driving metastatic potential [35]. Here, our findings also showed that NKD2 could function as a tumor suppressor and its overexpression impaired gastric cancer cells proliferation.

In conclusion, our study showed for the first time that lncRNA ZFAS1 expression is up-regulated in gastric cancer tissues and cells, and its overexpression is associated with poor prognosis and may be a negative prognostic factor for gastric cancer patients. Knockdown of ZFAS1 exerted tumor-suppressive functions through reducing cell proliferation and inducing cell apoptosis. Furthermore, ZFAS1 mediated the oncogenic effects is partially through its epigenetic silencing of the KLF2 and NKD2 expression by binding with PRC2 and LSD1. Our findings further the understanding of gastric cancer pathogenesis, and facilitate the development of lncRNA-directed diagnostics and therapeutics against this disease. However, whether ZFAS1 could regulate other possible targets and the mechanism that underlie regulatory behaviors were not investigated in this study, which needs to be further investigated.

## MATERIALS AND METHODS

### Microarray data analysis

Four human microarray datasets including GSE37023, GSE13911, GSE65801 and GSE51575 were obtained from public GEO database (http://www.ncbi.nlm.nih.gov/geo) and normalized using Robust Multichip Average (RMA). After probe sequences were downloaded from GEO or microarray manufacturers, blast+2.2.30 was used to re-annotates probe according to GENCODE Release 21 sequence databases for lncRNA.

### Clinical specimens and cell lines

Gastric cancer specimens and the corresponding adjacent noncancerous tissues were obtained from Jiangsu Province Hospital between 2010 and 2011 with informed consent. The patients were diagnosed with gastric cancer based on histopathological evaluation, and no local or systemic treatment was conducted before surgery. The protocols used in the study were approved by the Research Ethics Committee of Nanjing Medical University. BGC823, SGC7901, MGC803, AGS, HGC27 gastric cancer cell lines and a normal gastric epithelium cell line (GES-1) were purchased from the Shanghai Cell Bank of Chinese Academy of Sciences (Shanghai, China). BGC823, MGC803 cells were cultured in RPMI 1640; SGC7901, AGS and HGC27 were cultured in DMEM medium with 10% fetal bovine serum (FBS) (Invitrogen, Carlsbad, CA, USA). All cell lines were characterized by DNA fingerprinting analysis using short tandem repeat markers at the bank.

### RNA extraction and qPCR assays

Total RNA from specimens and cells was isolated with TRIzol reagent (Invitrogen) according to the manufacturer's instructions. 1μg RNA was reverse transcribed in a final volume of 20 μl using random primers under standard conditions for the PrimeScript RT reagent Kit (TaKaRa, Dalian, China). SYBR Premix Ex Taq (TaKaRa, Dalian, China) was used for Quantitative real-time PCR (qPCR) assays, which was carried out on Applied Biosystems 7500 Real-Time PCR System (Applied Biosystems). The specific primers used are presented in Supplementary Table S1. Our qPCR results were analyzed and expressed relative to threshold cycle (CT) values, and then converted to fold changes.

### Cell transfection

Human ZFAS1 transcript 1 cDNA and short-hairpin RNA directed against ZFAS1 was inserted into the pCDNA3.1 and pENTR™ H1 vector. Plasmid vectors (pCDNA-ZFAS1, sh-ZFAS1 and empty vectors) for transfection were prepared using DNA Midiprep or Midiprep kits (Qiagen, Hilden, Germany), and transfected into cells. The si-ZFAS1, si-EZH2, si-LSD1 or negative control siRNAs were used to knockdown their expression, and all siRNA and shRNA sequence were shown in Supplementary Table S1. GC cells were grown in 6-well plates and transfected by Lipofectamine 2000 (Invitrogen) according to the manufacturer's instructions. At 48 h post-transfection, cells were harvested for qPCR or western blot analysis.

### Cell proliferation assays

Cell proliferation ability was examined using a Cell Proliferation Reagent Kit I (MTT) (Roche Applied Science) and EdU assay kit (Life Technologies Corporation Carlsbad, CA, USA). Colony formation assays were performed to monitor GC cells cloning capability.

### Cell apoptosis assays

BGC-823 and SGC-7901 cells transfected with si-ZFAS1 or si-NC were harvested 48 hr after transfection by trypsinization. After the double staining with FITC-Annexin V and Propidium iodide (PI), the cells were analyzed with a flow cytometry (FACScan®; BD Biosciences) equipped with a CellQuest software (BD Biosciences).

### In vivo tumor formation assay

4 weeks female athymic BALB/c nude mice were maintained under specific pathogen-free conditions and manipulated according to protocols approved by the Shanghai Medical Experimental Animal Care Commission. sh-ZFAS1 or empty vector stably transfected BGC823 cells were harvested and. For tumor formation assay, 10^7^ cells was subcutaneously injected into a single side of each mouse. Tumor growth was examined every three days, and tumor volumes were calculated using the equation V = 0.5 × D × d2 (V, volume; D, longitudinal diameter; d, latitudinal diameter). This study was carried out in strict accordance with the recommendations in the Guide for the Care and Use of Laboratory Animals of the National Institutes of Health. The protocol was approved by the Committee on the Ethics of Animal Experiments of the Nanjing medical University.

### RNA immunoprecipitation

RNA immunoprecipitation was used to investigate whether ZFAS1 could interact or bind with the potential binding protein (EZH2, SUZ12, LSD1 and HuR et al.) in GC cells. We used the EZMagna RIP kit (Millipore, Billerica, MA, USA) following the manufacturer's protocol. BGC-823 and SGC-7901 cells were lysed in complete RIP lysis buffer, and the extract was incubated with magnetic beads conjugated with antibodies that recognized EZH2, SUZ12, LSD1 or control IgG (millipore) for 6hr at 4°C. Then, the beads were washed and incubated with Proteinase K to remove proteins. Finally, purified RNA was subjected to qRT-PCR analysis to demonstrate the presence of ZFAS1 using specific primers.

### RNA pull-down assays

ZFAS1 transcripts were transcribed using T7 RNA polymerase (Ambio life) in vitro, then by using the RNeasy Plus Mini Kit (Qiagen) and treated with DNase I (Qiagen). Purified RNAs were biotin-labeled with the Biotin RNA Labeling Mix (Ambio life). Positive control, negative control and Biotinylated RNAs were mixed and incubated with BGC823 cell lysates. Then, magnetic beads were added to each binding reaction, and incubated at room temperature. Finally, the beads were washed, and the eluted proteins were detected by western blot analysis.

### Chromatin immunoprecipitation

BGC-823 and SGC-7901 cells were treated with formaldehyde and incubated for 10 mins to generate DNA-protein cross-links. Cell lysates were then sonicated to generate chromatin fragments of 200-300 bp and immunoprecipitated with EZH2, LSD1 and H3K27me3 and H3K4me2-specific antibody (Millipore) or IgG as control. Precipitated chromatin DNA was recovered and analyzed by qRT-PCR.

### Subcellular fractionation location

The separation of nuclear and cytosolic fractions was performed using the PARIS Kit (Life Technologies) according to the manufacturer's instructions.

### Western blot assay and antibodies

BGC823 and SGC7901 cells were lysed with RIPA extraction reagent (Beyotime, Beijing, China) supplemented with a protease inhibitor cocktail (Roche, CA, USA) and phenylmethylsulfonyl fluoride (Roche). 40μg protein were separated by 10% SDS-polyacrylamide gel electrophoresis (SDS-PAGE), transferred to 0.22μm pvdf membranes (Millipore) and incubated with KLF2 (sigma), NKD2 (abcam) or GAPDH (abcam) antibodies. ECL chromogenic substrate was used to were quantified by densitometry (Quantity One software; Bio-Rad). GAPDH antibody was used as control.

### Statistical analysis

The Students t test (2 tailed), one-way ANOVA, and Mann-Whitney U test were conducted to analyze the in vitro and in vivo data by SPSS 17.0 software. P values less than 0.05 were considered significant.

## SUPPLEMENTARY MATERIALS FIGURES AND TABLES




