# Supplementary material for: Long noncoding RNA ZFAS1 promotes gastric cancer cells proliferation by epigenetically repressing KLF2 and NKD2 expression
Source: Oncotarget. 2016 May 26;8(24):38227–38. doi: 10.18632/oncotarget.9611 (PMC5503528; doi:10.18632/oncotarget.9611)
Supplement: Supplementary file 1 [file oncotarget-08-38227-s001.pdf]

Long noncoding RNA ZFAS1 promotes gastric cancer cells proliferation by epigenetically repressing KLF2 and NKD2 expression

SUPPLEMENTARY FIGURES AND TABLE

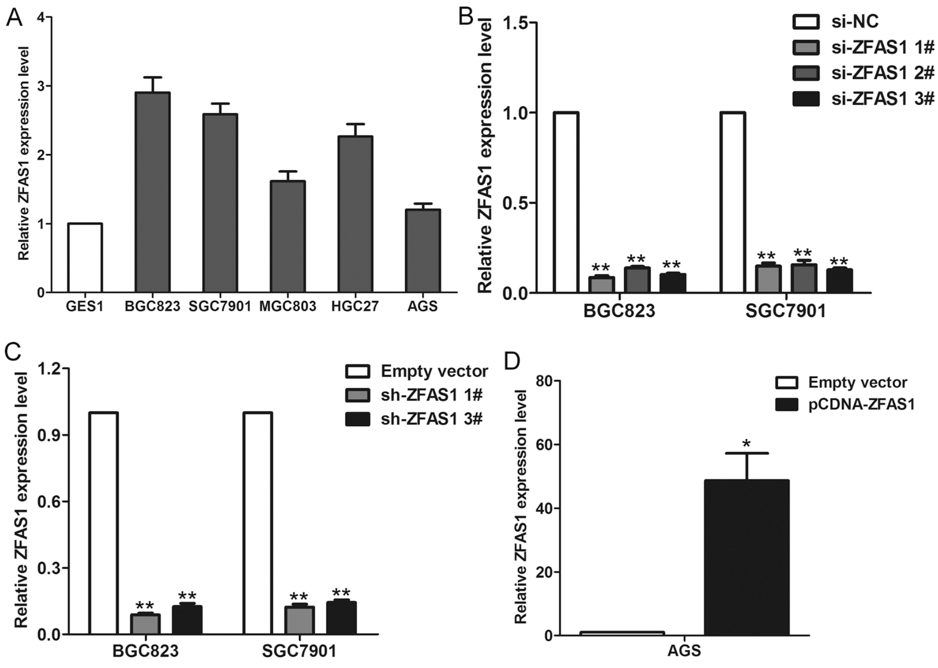

Supplementary Figure S1: A. ZFAS1 expression levels were detected by qPCR in gastric cancer cells lines. **B, C.** ZFAS1 expression levels were detected by qPCR in BGC823 and SGC7901 cells transfected with si-ZFAS1 and shZFAS1. **D.** ZFAS1 expression levels were detected by qPCR in AGS cells transfected with ZFAS1 overexpression vector. *P < 0.05 and **P < 0.01

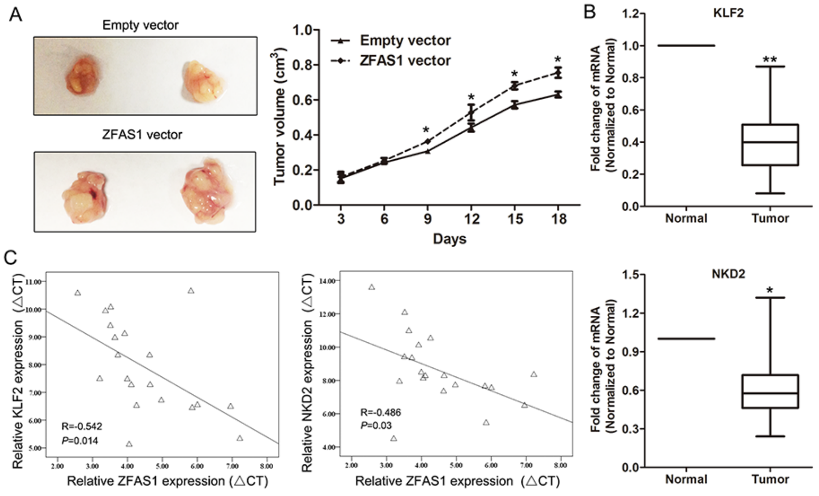

Supplementary Figure S2: A. The stable ZFAS1 overexpression AGS cells were used for the in vivo assays. The tumors from two groups nude mice were shown and tumor growth curves were measured and shown after the injection of AGS cells. The tumor volume was calculated every 3 days. **B.** KLF2 and NKD2 expression levels were detected by qPCR in gastric cancer tissues. **C.** The relationship between ZFAS1 expression and KLF2, NKD2 in gastric cancer tissues were analyzed. *P < 0.05 and **P < 0.01

Supplementary Table S1: primer, siRNA and shRNA sequence and Antibody information.

See Supplementary Table 1
